# A Comparative Analysis of the Antimicrobial Efficacy of Nisin in Different Vehicles Against Enterococcus faecalis: An In Vitro Study

**DOI:** 10.7759/cureus.66204

**Published:** 2024-08-05

**Authors:** Gaurav Patri, Ishika Chatterjee, Harshita Lath, Yash Sinha, Pratik Agrawal, Neelanjana Majee, Sonali Bansal

**Affiliations:** 1 Department of Conservative Dentistry and Endodontics, Kalinga Institute of Dental Sciences, Kalinga Institute of Industrial Technology (KIIT) Deemed to be University, Bhubaneswar, IND

**Keywords:** enterococcus faecalis (e. faecalis), nisin, direct contact test, chlorhexidine, chitosan, antimicrobial efficacy

## Abstract

Aim

To evaluate and compare the antimicrobial efficacy of nisin in different carriers against *Enterococcus faecalis*.

Materials and methods

Test materials were divided into four groups of five samples each as follows: group 1 = nisin + 17% ethylenediaminetetraacetic acid (EDTA); group 2 = nisin + 2% chitosan; group 3 = nisin + 2% chlorhexidine; group 4 = nisin + distilled water (control). The antimicrobial effectiveness was assessed using the direct contact method, where a standardized *E. faecalis* suspension was applied to the test materials. Optical density (OD) was assessed using enzyme-linked immunosorbent assay (ELISA) at the end of days one and seven. Data were analyzed using ANOVA and Tukey’s post hoc analysis. The level of significance was set at p < 0.05.

Results

On day one, there was a significant difference in the mean OD values (p < 0.001) with group 3 showing the highest, followed by groups 1, 2, and 4. On day seven, all groups demonstrated antibacterial activity (group 1 > group 3 > group 4 > group 2) but the differences were not statistically significant (p = 0.393). Intragroup analysis showed a decrease in the OD values from day one to day seven, the difference of which was not significant in all groups except group 1, which showed a significant difference (p = 0.035).

Conclusion

The antibacterial efficacy of nisin was synergistically enhanced with the addition of 17% EDTA and 2% chlorhexidine over seven days against *E. faecalis*.

## Introduction

The efficacy of treatment for endodontic infections hinges on the successful eradication of the causative microorganisms [1]. The predominant techniques employed involve the mechanical manipulation of instruments and the use of antibacterial solutions for irrigation. These methods aim to eliminate intraradicular biofilms that have affixed themselves to dentin walls. Despite these efforts, a notable challenge persists, as the limitations of endodontic instruments prevent access to every dentin wall. Furthermore, contemporary antimicrobial treatments fall short in completely eradicating biofilms, resulting in persistent infection even following root canal preparation [2].

*Enterococcus faecalis*, the predominant facultative anaerobic bacteria identified in both secondary and persistent root canal infections, thrives in anatomical complexities, posing a formidable clinical challenge for complete eradication. Therefore, achieving healing in infected root dentin necessitates a combination of antimicrobial medicaments and mechanical cleansing to enhance treatment success [3].

A frequently employed intracanal medicament is calcium hydroxide; however, numerous studies indicate its ineffectiveness against *E. faecalis*, an organism known for its tolerance to various growth conditions. Furthermore, the low solubility and diffusibility of Ca(OH)2 may hinder its ability to penetrate dentinal tubules and exert any significant action [1,4].

Nisin, a cationic antibacterial peptide produced by lactic acid bacteria, disturbs anionic biofilm surfaces by disrupting the bacterial cell membrane [5]. It exhibits high efficacy against multidrug-resistant *E. faecalis* isolates, gram-positive bacteria, and their spores [6]. Remarkably, nisin maintains its antibacterial activity and stability at low pH values. However, Sebti et al. demonstrated that the solubility and stability of nisin diminish its antibacterial effectiveness when the pH exceeds 4 [7]. Consequently, supplementary chemicals are required to ensure prolonged action [7]. As an intracanal medicament, nisin is commonly blended with a vehicle to facilitate its application. The choice of vehicle directly influences the concentration and rate of ionic liberation, as well as the antibacterial efficacy when the paste is introduced into the contaminated area [8].

Chlorhexidine (CHX) has found extensive application in endodontics, demonstrating antibacterial efficacy against both gram-positive and gram-negative microorganisms. Its substantivity property, adsorption capacity, and gradual release of active molecules by dental tissue make it suitable for use as a vehicle. The substantiative antimicrobial activity of chlorhexidine proves effective in the complete elimination of *E. faecalis* from dentinal tubules for up to 15 days [9].

In recent investigations, chitosan has been scrutinized as a potential vehicle in endodontics. As an aminopolysaccharide derived from the partial deacetylation of chitin, chitosan possesses noteworthy attributes, including high bioactivity, biocompatibility, hydrophilicity, chelating capabilities, and antibacterial activity. Its effectiveness has been demonstrated against various oral microorganisms, including *Candida albicans*, *Streptococcus mutans*, and *E. faecalis* [10].

Nisin exhibits meager activity against a biofilm model predominantly composed of gram-negative anaerobic species, with the outer membrane of these organisms potentially impeding the antimicrobial peptide's approach to lipid II in the inner membrane. To address this challenge, combining nisin with ethylenediaminetetraacetic acid (EDTA) has been suggested [11,12].

EDTA, a cation chelator, demonstrates the capability to destabilize the outer membrane of gram-negative bacteria, potentially augmenting the activity of other antimicrobials. Furthermore, it contributes to the reduction of biofilm matrix strength by sequestering cations, leading to increased detachment of bacterial cells from the biofilm [11,12].

The application of nisin as a medicament has yielded promising outcomes and has been assessed in conjunction with various vehicles [11,12]. Notably, there is a dearth of literature comparing the antibacterial efficacy of nisin in combination with different vehicles. Thus the aim of the study was to evaluate the antimicrobial efficacy of nisin in various vehicles against *E. faecalis*.

The null hypothesis suggested that using various vehicles with nisin does not result in any differences in its antibacterial effectiveness.

## Materials and methods

Preparation and formulation of medicaments

Commercially available nisin powder (Delta Chemsol Pharmaceuticals, Mumbai, India) was dissolved in each vehicle to a concentration of 100 mg/ml [1]. Chitosan 2% was prepared by dissolving 2 g of chitosan in 100 ml of water with five drops of acetic acid added to it. The mixture was blended for two hours using a magnetic stirrer [1]. Commercially available solutions of 17% EDTA (Prevest DenPro, Jammu, India) and 2% chlorhexidine (Hexachlor, SafeEndo, Vadodara, India) were used.

**Figure 1:**
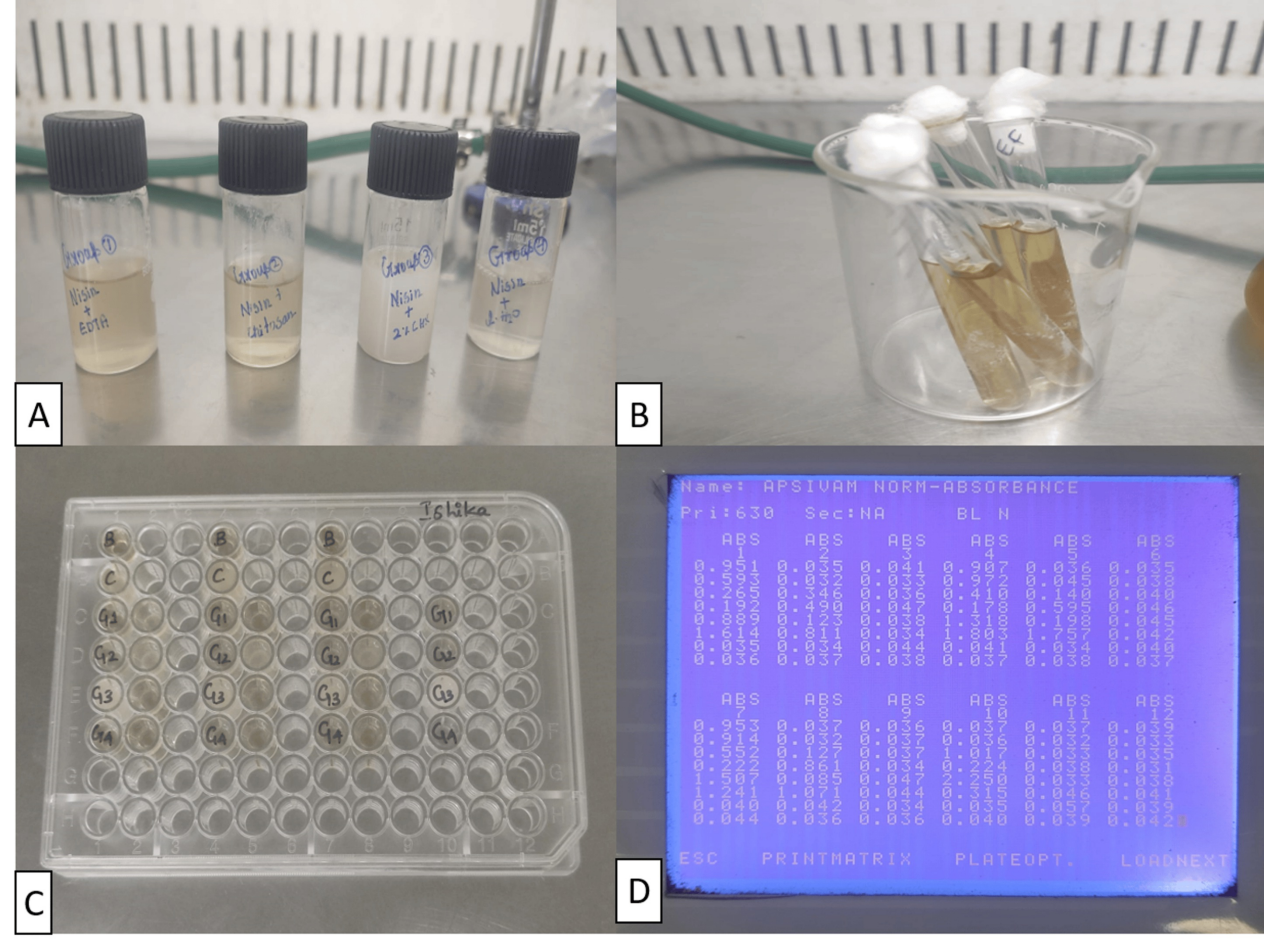
Pictorial representation of the experimental procedure. A: Test sample preparation. B: *E. faecalis* culture. C: Direct contact test being carried out in 96-well microtiter plates. D: Optical density reading in the enzyme-linked immunosorbent assay (ELISA) machine.

This study was conducted on four primary groups outlined as follows (Figure 1A): group 1: nisin + 17% EDTA mixed in a 1:1 ratio; group 2: nisin + 2% chitosan mixed in a 1:2 ratio; group 3: nisin + 2% CHX mixed in 1:1 ratio; group 4: nisin + distilled water (control).

Test microorganism

*Enterococcus faecalis* (American Type Culture Collection (ATCC) 29212) was initially grown on a brain heart infusion (BHI) agar plate and then transferred to a nutrient agar medium for further cultivation. After confirming the purity of the strain, a bacterial suspension was prepared in 5 ml of 85% saline and adjusted to 90% transmittance at 800 nm using a spectrophotometer, aligning with a 0.5 McFarland standard (approximately 1.5 x 10^8 CFU) (Figure 1B) [13].

Direct contact test

The evaluation of antimicrobial efficacy in this study employed the direct contact test, focusing on assessing bacterial growth turbidity in 96-well microtiter plates [13]. In this approach, freshly mixed test materials were positioned at the base of four wells in the microtiter plate at a 2 mm height and exposed to a 10 μl bacterial suspension (Figure 1C) [13]. Following direct contact, 245 μl of BHI broth was introduced, and after a two-minute mixing interval, 15 μl of the resulting mixture was transferred into four adjacent wells with 215 μl of fresh medium [13]. Continuous monitoring of bacterial expansion kinetics in each well was conducted using an enzyme-linked immunosorbent assay (ELISA) reader set to 630 nm (Figure 1D) [13]. Densitometric values were recorded on both the first and seventh day for each sample set. To ensure robustness, the experiments were replicated three times [13].

Statistical analysis

The data were analyzed using IBM SPSS version 25 (IBM Corp., Armonk, NY). Day one and day seven values have been presented as mean ± standard deviation and compared among the four groups using ANOVA, followed by Tukey’s post-hoc analysis for significant results. The level of significance was set at p < 0.05.

## Results

Table 1 depicts the intergroup comparison of optical density (OD) values among the four groups after day one. All groups demonstrated antibacterial activity. There was a significant difference in the mean OD values among the groups (p < 0.001). The values were highest for group 3, followed by groups 1, 2, and 4. A further post-hoc analysis (Table 2) revealed significant differences (p < 0.001) between each group compared against each other, except for between groups 1 and 3 (p = 0.529), which was not significant.

**Table 1:** Inter-group comparison of day one values among the four groups. S: significant; EDTA: ethylenediaminetetraacetic acid; CHX: chlorhexidine.

Group	Mean	Standard deviation	Minimum	Maximum	P-value
1 (nisin + EDTA)	82.00	13.85	66	90	<0.001, S
2 (nisin + 2% chitosan)	51.33	5.68	45	56	
3 (nisin + 2% CHX)	91.67	0.57	91	92	
4 (nisin + distilled water)	22.67	7.63	16	31	

**Table 2:** Post-hoc comparison of significant day one values. S: significant; NS: non-significant.

Group 1	Group 2	Mean difference	P-value
1	2	-30.66	0.009, S
	3	9.66	0.529, NS
	4	-59.33	<0.001, S
2	3	40.33	0.002, S
	4	-28.66	0.013, S
3	4	-69.00	<0.001, S

Table 3 depicts the intergroup comparison of OD values among the four groups after day seven. All groups demonstrated antibacterial activity. The values were highest for group 1, followed by groups 3, 4, and 2. These differences were not statistically significant (p = 0.393).

**Table 3:** Inter-group comparison of day seven values among the four groups. NS: non-significant; EDTA: ethylenediaminetetraacetic acid; CHX: chlorhexidine.

Group	Mean	Standard deviation	Minimum	Maximum	P-value
1 (nisin + EDTA)	79.00	13.000	64	87	0.393, NS
2 (nisin + 2% chitosan)	34.00	18.028	14	49	
3 (nisin + 2% CHX)	59.33	42.911	10	88	
4 (nisin + distilled water)	43.33	42.336	15	92	

Table 4 depicts the comparison of OD values between the groups on day one and day seven. There was a decrease in the OD values from day one and day seven, the difference of which was not significant in all groups, except group 1, which showed a significant difference (p = 0.035).

**Table 4:** Comparison of day one and day seven values in each of the four groups. EDTA: ethylenediaminetetraacetic acid; CHX: chlorhexidine; S: significant; NS: non-significant.

Group	Day 1	Day 7	P-value
1 (nisin + EDTA)	82.00	79.00	0.035, S
2 (nisin + 2% chitosan)	51.33	34.00	0.156, NS
3 (nisin + 2% CHX)	91.67	59.33	0.325, NS
4 (nisin + distilled water)	22.67	43.33	0.499, NS

## Discussion

*E. faecalis* stands out as one of the most antibiotic-resistant bacteria currently recognized. This resilience is attributed to its rapid acquisition and dissemination of antibiotic-resistance genes through pheromone prompts generated within its own genus and species as well as signals from other bacterial genera [14]. The extensive use of antibiotics over time has exacerbated bacterial resistance, particularly in the context of treating oral diseases, leading to the emergence of multidrug-resistant microorganisms. In response to this challenge, there is a growing exploration of local antibiotic applications as a strategy to mitigate drug resistance, minimize systemic complications, and enhance therapeutic efficacy [15]. Among many, nisin is found to be a promising agent in eliminating *E. faecalis* when used as an intracanal medicament [1,3].

*E. faecalis* (ATCC 29212) is commonly employed in survival and biofilm studies, given its extensive use as an illustrative control strain in laboratory and clinical experiments [14]. Accordingly, *E. faecalis* was selected as the test organism for the present study.

This study utilized the direct contact test method as described by Weiss et al., which ensures direct interaction between the test organism and the material being assessed, regardless of the antimicrobial components' solubility and diffusibility [16]. This approach allows for the evaluation of water-insoluble substances under diverse conditions, including aging [16]. Importantly, the direct contact test is a dependable and consistent qualitative technique that minimizes confounding variables and remains unaffected by the inoculum size in contact with the test material [13].

The primary aim of this study was to evaluate the effect on the antibacterial efficacy of nisin against *E. faecalis* when mixed with various vehicles. The obtained results led to the rejection of the null hypothesis, indicating variations in the antibacterial efficacy among the test agents.

The present study showed that nisin with any vehicle demonstrated antibacterial activity from day one (Table 1). Group 3 (nisin + 2% CHX) and group 1 (nisin + 17% EDTA) performed better than group 2 (nisin + 2% chitosan) and group 4 (nisin + distilled water). Although intergroup comparison showed significant results (p < 0.001), groups 3 and 1, did not show significant differences (Table 2). On day seven, although group 1 performed better, all groups demonstrated antibacterial activity with non-significant differences (Table 3).

Nisin demonstrates its antibacterial efficacy by integrating into the bacterial plasma membrane and activating bacterial murein hydrolases, leading to the impairment or degradation of peptidoglycans and subsequent cell lysis. Research indicates its interaction with the phospholipid membrane of the target bacterial cell, resulting in autolysis and irreversible damage to the plasma membrane. Additionally, nisin disrupts cellular mechanisms, prompting the leakage of small intracellular contents from the cell [3].

EDTA, a widely used chelating agent, eliminates dentinal debris from the smear layer formed during mechanical instrumentation. A noteworthy discovery in a study revealed that 17% EDTA facilitated bacterial reduction in mature biofilms, comparable to the effects of 1.5% NaOCl treatment. The antibiofilm efficacy of EDTA is attributed to its capacity to disengage cells within the biofilm, achieved through the undermining of the biofilm matrix via cation sequestration. Furthermore, EDTA's cation-chelating properties can impede bacterial growth by destabilization of the outer membrane of gram-negative bacteria, providing insight into its notable antibacterial effectiveness against *E. faecalis* [11,12].

Chlorhexidine is widely acknowledged as the gold standard due to its remarkable antibacterial efficacy against *E. faecalis* [17,18]. As a cationic biguanide and antiseptic, it possesses substantive properties and exhibits minimal toxicity even at higher concentrations. At lower concentrations, it operates in a bacteriostatic manner, while at higher concentrations, it demonstrates bactericidal activity, displaying antimicrobial effectiveness from concentrations as low as 0.1%. Notably, chlorhexidine's bactericidal activity is evident at 2%, and it maintains biocompatibility. The minimum inhibitory concentration (MIC) of chlorhexidine against *E. faecalis* is recorded at 0.156% [3].

The present study showed that nisin when mixed with either EDTA or chlorhexidine had a probable synergistic effect that could account for its improved antibacterial activity when compared to its counterparts. The addition of 2% chitosan to nisin has demonstrated good bactericidal activity against *E. faecalis*, which is similar to the findings of Kristl et al. [19] and Harshitha et al. [1]. In our study, the synergism of nisin with chitosan was found to be lesser than EDTA and chlorhexidine.

Distilled water is a neutral vehicle with no antimicrobial properties and creates an alkaline pH for a short time period [1,3]. This could have been attributed to the poor performance of nisin with distilled water, which is also in accordance with Harshitha et al. [1].

This investigation sought to assess experimental medicaments after one day, exploring the potential for superior antibacterial efficacy compared to medicaments recommended for periods exceeding one week (> seven days). Additionally, the extended presence of medicaments in the canal may compromise their effectiveness. Emphasizing the critical role of time duration in antimicrobial activity, our study observed a decline in mean OD values of test agents over seven days compared to the one-day measurement. Comparable results were reported by Harshitha et al. [1] when nisin was combined with chitosan and distilled water [1].

From a limitations perspective, this study focused on a single microorganism and does not truly reflect the complex ecosystem of the roots. Furthermore, the effect of the medicaments on the root dentin was not investigated. Future in vitro/in vivo studies in this regard and with a polymicrobial biofilm can be undertaken. In vivo studies will help in understanding the real-world applicability of the medicament and the effectiveness of the medicaments when exposed to polymicrobial biofilm and host immune response. Additionally, it can help identify any potential side effects on the surrounding tissues and overall oral health.

## Conclusions

The antibacterial efficacy of nisin was enhanced with the addition of 17% EDTA, 2% chlorhexidine, and 2% chitosan, which was evident from day one but gradually decreased by day seven. Overall, the synergism of 17% EDTA or 2% chlorhexidine with nisin was more efficacious against *E. faecalis*.

## References

[1] Harshitha VS, Ranjini MA, Nadig RR (2022). Antibacterial efficacy of nisin, calcium hydroxide, and triple antibiotic paste in combination with chitosan as an intracanal medicament against Enterococcus faecalis - an in vitro study. J Conserv Dent.

[2] Siqueira JF Jr, Rôças IN (2008). Clinical implications and microbiology of bacterial persistence after treatment procedures. J Endod.

[3] Tripathi S, Mittal P, Deb S, Verma S (2019). In vitro evaluation of antibacterial efficacy of nisin calcium hydroxide and triple antibiotic paste in three different vehicle. J Med Sci Clin Res.

[4] Haapasalo HK, Sirén EK, Waltimo TM, Ørstavik D, Haapasalo MP (2000). Inactivation of local root canal medicaments by dentine: an in vitro study. Int Endod J.

[5] Mathur H, Field D, Rea MC, Cotter PD, Hill C, Ross RP (2018). Fighting biofilms with lantibiotics and other groups of bacteriocins. NPJ Biofilms Microbiomes.

[6] Garcia-Gutierrez E, O'Connor PM, Saalbach G (2020). First evidence of production of the lantibiotic nisin P. Sci Rep.

[7] Sebti I, Chollet E, Degraeve P, Noel C, Peyrol E (2007). Water sensitivity, antimicrobial, and physicochemical analyses of edible films based on HPMC and/or chitosan. J Agric Food Chem.

[8] Robert GH, Liewehr FR, Buxton TB, McPherson JC 3rd (2005). Apical diffusion of calcium hydroxide in an in vitro model. J Endod.

[9] Bilgi PS, Shah NC, Mehta J (2017). Comparative evaluation of mixture of calcium hydroxide and chlorhexidine, with triple antibiotic paste and combination of calcium hydroxide, chlorhexidine, and lycopene on incidence of interappointment flare-up: an in vivo study. Int J Clin Dent Res.

[10] Ballal N, Kundabala M, Bhat K, Acharya S, Ballal M, Kumar R, Prakash P (2009). Susceptibility of Candida albicans and Enterococcus faecalis to chitosan, chlorhexidine gluconate and their combination in vitro. Aust Endod J.

[11] Pinheiro ET, Karygianni L, Attin T, Thurnheer T (2021). Antibacterial effect of sodium hypochlorite and EDTA in combination with high-purity nisin on an endodontic-like biofilm model. Antibiotics.

[12] Yüksel FN, Buzrul S, Akçelik M, Akçelik N (2018). Inhibition and eradication of Salmonella typhimurium biofilm using P22 bacteriophage, EDTA and nisin. Biofouling.

[13] Ghatole K, Patil A, Giriyappa RH, Singh TV, Jyotsna SV, Rairam S (2016). Evaluation of antibacterial efficacy of MTA with and without additives like silver zeolite and chlorhexidine. J Clin of Diagn Res.

[14] Tong Z, Zhang Y, Ling J, Ma J, Huang L, Zhang L (2014). An in vitro study on the effects of nisin on the antibacterial activities of 18 antibiotics against Enterococcus faecalis. PLoS One.

[15] Khan M, Samant PS, Chauhan R, Khan R, Siddiqui S, Sachan S (2023). Comparative evaluation of effectiveness of nisin, amoxicillin/clavulanic acid (Augmentin) and chlorhexidine on E. faecalis as an intracanal irrigant: an in-vitro study. J Pharm Bioallied Sci.

[16] Weiss EI, Shalhav M, Fuss Z (1996). Assessment of antibacterial activity of endodontic sealers by a direct contact test. Endod Dent Traumatol.

[17] Gomes BP, Ferraz CC, Vianna ME, Berber VB, Teixeira FB, Souza-Filho FJ (2001). In vitro antimicrobial activity of several concentrations of sodium hypochlorite and chlorhexidine gluconate in the elimination of Enterococcus faecalis. Int Endod J.

[18] Gomes BP, Vianna ME, Zaia AA, Almeida JF, Souza-Filho FJ, Ferraz CC (2013). Chlorhexidine in endodontics. Braz Dent J.

[19] Kristl J, Šmid-Korbar J, Štruc E, Schara M, Rupprecht H (1993). Hydrocolloids and gels of chitosan as drug carriers. Int J Pharm.

